# In Vitro Investigation of Wear of CAD/CAM Polymeric Materials Against Primary Teeth

**DOI:** 10.3390/ma10121410

**Published:** 2017-12-09

**Authors:** Jae-Won Choi, Eun-Ju Song, Jong-Hyun Shin, Tae-Sung Jeong, Jung-Bo Huh

**Affiliations:** 1 Department of Prosthodontics, Dental Research Institute, Institute of Translational Dental Sciences, BK21 PLUS Project, School of Dentistry, Pusan National University, Yangsan 50612, Korea; won9180@hanmail.net; 2 Department of Pediatric Dentistry, School of Dentistry, Pusan National University, Yangsan 50612, Korea; behana@hanmail.net (E.-J.S.); newbeline@daum.net (J.-H.S.); tsjeong@pusan.ac.kr (T.-S.J.)

**Keywords:** CAD/CAM, resin nano ceramic, polymer infiltrated ceramic network, PEKK, primary tooth, wear

## Abstract

The aim of the study was to evaluate the effects of polymeric computer-aided design/computer-aided manufacturing CAD/CAM materials on antagonistic primary tooth wear. Five CAD/CAM polymeric materials were examined: Vipi Block Monocolor (VBM), Yamahachi polymethylmethacrylate (PMMA) (YAP), Mazic Duro (MZD), Vita Enamic (ENA), and Pekkton (PEK). All of the specimens were tested in a thermomechanical loading machine with the primary canine as the antagonist (50 N, 1.2 × 10^5^ cycles, 1.7 Hz, 5/55 °C). The wear losses of the antagonist tooth and the restorative materials were calculated using reverse modelling software and an electronic scale. VBM and ENA showed significantly higher antagonist tooth wear than PEK (*p* < 0.05), but there was no significant difference observed among VBM, YAP, MZD, and ENA (*p* > 0.05). PEK showed the largest value in both material volumetric and weight losses. In terms of material volumetric losses, there was no significant difference between all of the groups (*p* > 0.05). In terms of material weight losses, PEK was significantly larger than ENA (*p* < 0.05), but there was no significant difference between VBM, YAP, MZD, and ENA (*p* > 0.05). Volumetric and weight losses of materials showed similar wear behaviour. However, the wear patterns of antagonists and materials were different, especially in PEK.

## 1. Introduction

Computer-aided design/computer-aided manufacturing (CAD/CAM) technology was introduced to the dental field in the 1980s, and over the past decade, its importance and popularity have rapidly increased. CAD/CAM dental restorations meet the requirements of standardised manufacturing processes that ensure uniform quality and restoration reproducibility [1]. As the demand for non-metallic restorations continues to increase in the dental field, several CAD/CAM polymers have been introduced as alternatives for ceramics with faster and lower cost-processing characteristics [2,3].

Ceramics are biocompatible, strong, aesthetically pleasing, and mimic the structural characteristics of teeth [4], but they also exhibit high fracture resistance and low material wear [5]. However, due to the abrasive effect and brittleness of ceramic, failure rates are high [6]. On the other hand, polymers have low elasticity moduli, which allow them to absorb stresses by deformation [7]. Previous studies on the wear behaviour of CAD/CAM polymers and ceramics have reported that the polymers cause less antagonistic enamel wear and cracking than ceramics [7,8]. However, polymeric CAD/CAM materials, such as composites and polymethylmethacrylate (PMMA)-based materials are inferior to ceramics in terms of material loss, biocompatibility, and mechanical properties [9]. As a result, a variety of polymer materials have been introduced that overcome these shortcomings and combine aesthetic and functional properties [10,11].

Ceramic–polymer composites combine the advantages of ceramics and composites [12]. Mazic Duro (Vericom, Anyang, Korea) and Vita Enamic (VITA Zahnfabrik, Bad Säckinge, Germany) are representative examples of such materials. Mazic Duro (MZD) is a resin/nano ceramic composite consisting of 80 wt % ceramic and 20 wt % resin matrix. The manufacturers of these products claim that ceramic polymer composite materials exhibit the advantages of high elasticity and malleability due to the presence of the reinforcing matrix, as well as the advantages of ceramics, that is, strength, resistance to discoloration, and aesthetics. Vita Enamic (ENA) has a structure similar to that of a polymer-infiltrated ceramic network. Its dominant ceramic is reinforced by a polymer network, and it has an interpenetrating network of appropriately incorporated ceramic and composite resin [13]. Materials with microstructures composed of interpenetrating networks have been reported to exhibit high flexural strengths and strains at failure [14], and have properties similar to those of natural teeth [11]. 

The high performance thermoplastic polyetherketoneketone (PEKK), a member of the polyaryletherketone (PAEK) polymer family, was recently introduced as a dental material. High performance polymers are considered dental materials that can replace metal and glass ceramics due to their acceptable fracture resistances, excellent stress distributions, and shock absorbing abilities [15]. In the dental field, PAEK polymers are used for temporary implant abutments, implant-supported bars, dental implants, dental clasps, and as frameworks for removable partial dentures [16,17]. Due to its wide processing parameters, PEKK can be used to fabricate crowns and fixed dental prostheses (FDPs) [18].

Primary tooth wear is a common phenomenon caused by the loss of enamel and dentin from occlusal surfaces [19]. Furthermore, the abrasiveness of primary and permanent teeth differ, presumably due to different physical characteristics [20], morphologies [21], and biting forces [22]. In a comparative study on the wear of primary and permanent teeth, it was reported that tooth wear was greater for primary teeth than permanent teeth, and that incisal/occlusal surfaces were most affected by tooth wear for both permanent and primary teeth [23]. Stainless steel crowns (SSCs) provide one of the most effective methods for restoring primary teeth, but are non-aesthetic [24]. In recent years, developments of CAD/CAM systems in pediatric dentistry have been applied to CAD/CAM ceramics for restorative restoration [25]. However, some of these CAD/CAM materials require additional processing after milling and specific equipment for firing and glazing [10]. In contrast, CAD/CAM polymeric materials can be produced easily and quickly using devices suitable for use in dental offices, and do not require additional processing [10].

The purpose of this study is to provide information on the possible use of various CAD/CAM materials for primary teeth restoration based on evaluations of wear relationships between CAD/CAM polymeric resins and primary teeth.

## 2. Materials and Methods

### 2.1. Fabrication of the Antagonists

**Figure 1:**
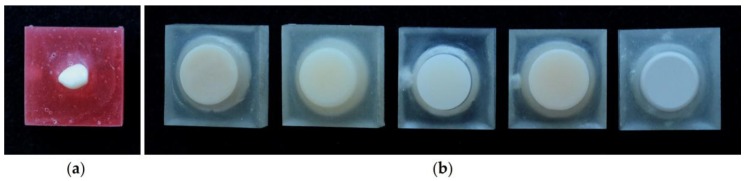
Preparation of the specimens. (**a**) Antagonistic primary teeth; (**b**) Materials specimens (from left, Vipi Block Monocolor: VBM, Yamahachi polymethylmethacrylate (PMMA): YAP, Mazic Duro: MZD, Vita Enamic: ENA and Pekkton: PEK).

The antagonists used in this study were primary canines with no cusp wear that were naturally dropped during transition to permanent dentition. They were collected under the approval of the Institutional Review Board of Pusan National University Dental Hospital (IRB no. PNUDH-2017-029). The collected teeth were washed with ultrasonic cleaner before use and stored in deionised water at 37 °C for 24 h. The cusps of the primary canine teeth were exposed by 5 mm using a mold of height 10 mm, width 20 mm, and depth 10 mm, and fixed using an acrylic resin (Orthodontic resin, Dentsply, PA, USA). Severely worn or broken teeth and teeth with caries were excluded (Figure 1a).

### 2.2. Fabrication of the Material Specimens

In this study, five CAD/CAM polymeric resin blocks were used: two of PMMA-based CAD/CAM materials: Vipi Block Monocolor (VBM) and Yamahachi PMMA (YAP), two of ceramic–polymer composite materials: Mazic Duro (MZD) and Vita Enamic (ENA), and one of a high performance polymer: Pekkton (PEK) (Table 1). A cylindrical stereolithography (STL) file (diameter 11 mm and height 13 mm) was designed by AutoCAD. The generated STL file was transferred to a milling machine to prepare 10 cylindrical specimens for each material. Specimens were ultrasonically cleaned in distilled water, and fixed with acrylic resin using a uniform mold in the same manner as antagonists (Figure 1b). All of the specimens were produced by a single dental technician.

### 2.3. Wear Simulation

**Figure 2:**
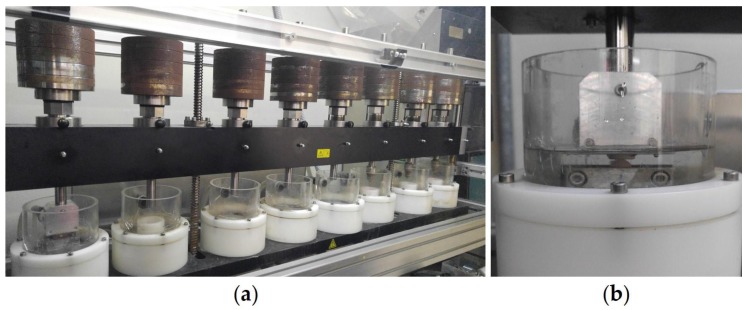
Set-up wear simulation. (**a**) Chewing simulator; (**b**) Specimens fixed in test chamber.

Material specimens and antagonists were mounted on a computer-supported chewing simulator (Chewing Simulator CS-4.8, SD Mechatronics, Feldkirchen-Westerham, Germany) (Figure 2a). The specimens were tested using a vertical load of 50 N, a frequency of 1.7 Hz, and a sliding movement of 0.7 mm for 120,000 cycles [26,27]. During the wear test, thermal stress was applied in distilled water at temperatures of 5 °C and 55 °C using 60 s cycles (Figure 2b).

### 2.4. Wear Measurements

**Figure 3:**
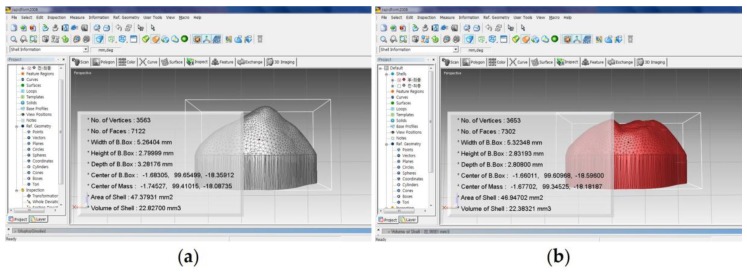
Volume measurements using a three-dimensional (3D) scanned image. (**a**) Before wear; (**b**) after wear.

Three-dimensional (3D) data of antagonists and material specimens were obtained before and after the experiment using a blue-light scanner (Identica blue, Medit, Seoul, Korea). The Model Creator module of the CAD software (Exocad Dental CAD, Exocad GmbH, Darmstadt, Germany) was used to delete 3D data corresponding to non-cusp areas of antagonists before and after the experiment to reduce errors in the scanning process. For this purpose, the 3D data of the antagonists before the experiment were placed in the position where only the exposed cusp areas of antagonists could be obtained from the CAD software (Exocad Dental CAD, Exocad GmbH, Darmstadt, Germany). Afterwards, the 3D data of the antagonists from after the experiment were imported and superimposed onto the 3D data of the antagonists from before the experiment. As a result, the two antagonists were placed in the same position on the CAD software, and the 3D data from before and after experiment corresponding to the cusp tip of the canine were obtained using the model creator module. Volumetric losses were analysed by importing newly obtained 3D data from reverse modelling software (RapidForm 2006; INUS Technology, Seoul, Korea) (Figure 3). Material specimens were also measured in the same manner as above. In addition, weight losses of material specimens before and after testing were measured using an electronic scale (Pioneer, Ohaus Co., Parsippany, NJ, USA).

### 2.5. Scanning Electron Microscopy (SEM)

To qualitatively evaluate the wear patterns of the antagonists and materials, specimens were observed by scanning electron microscopy (S-3500, Hitachi Ltd., Tokyo, Japan) at magnifications of 35×, 150×, and 1000× at 15 keV.

### 2.6. Statistics

The significance of changes in tooth volumes and material weights were evaluated using SPSS (version 23.0, IBM Corp, Armonk, NY, USA). The Shapiro–Wilk test and Levene’s test were used to check distribution normality and variance homogeneity. Data were analysed using the Kruskal–Wallis test, followed by the Mann–Whitney U-test using Bonferroni’s correction (α = 0.05/10 = 0.005). Statistical significance was accepted at *p* values < 0.05.

## 3. Results

### 3.1. Wear Loss

After the experiment, the mean values and standard deviations of volume changes of the antagonists (V_a_), restorative materials (V_m_), materials and corresponding antagonists (V_t_), and weight changes of materials (V_w_) in each group are shown in Table 2. VBM (1.4261 ± 1.6156 mm^3^) showed the greatest amount of antagonist wear, followed in decreasing order by ENA (1.3983 ± 0.9264 mm^3^), YAP (1.2833 ± 1.9111 mm^3^), MZD (0.7260 ± 0.5786 mm^3^), and PEK (0.2621 ± 0.2707 mm^3^). The mean volume losses in the VBM and ENA groups were significantly greater than those in the PEK group (*p* < 0.05), but there was no significant intergroup difference observed between the VBM, YAP, MZD, and ENA groups (*p* > 0.05). PEK (1.7617 ± 1.4097 mm^3^) showed the most material volume losses, followed by VBM (1.0306 ± 0.8135 mm^3^), YAP (0.8857 ± 0.5807 mm^3^), MZD (0.7432 ± 0.6296 mm^3^), and ENA (0.7197 ± 0.4958 mm^3^), but no significant intergroup difference was found (*p* > 0.05). VBM (2.4567 ± 1.9720 mm^3^) showed the most total wear, followed in decreasing order by YAP (2.1690 ± 1.7496 mm^3^), ENA (2.1180 ± 1.2189 mm^3^), PEK (2.0238 ± 1.4974 mm^3^), and MZD (1.4692 ± 0.6006 mm^3^), but no significant difference was observed between groups (*p* > 0.05). In terms of material weight losses, PEK (0.0008 ± 0.0003 g) lost the most, followed by YAP (0.0006 ± 0.0005 g), VBM (0.0005 ± 0.0002 g), MZD (0.0005 ± 0.0002 g), and ENA (0.0003 ± 0.0003 g). The PEK group also lost significantly more material than the ENA group (*p* < 0.05), but no significant difference was observed between weight losses in the VBM, YAP, MZD, and ENA groups (*p* > 0.05).

### 3.2. Scanning Electron Microscopy (SEM)

**Figure 4:**
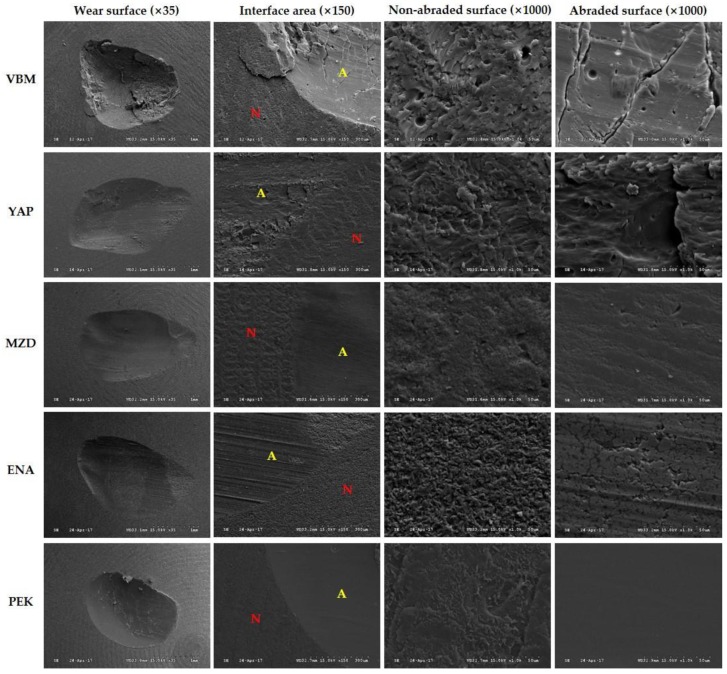
Scanning electron micrographs of restorative material specimens after 120,000 chewing cycles (VBM: Vipi Block Monocolor; YAP: Yamahachi PMMA; MZD: Mazic Duro; ENA: Vita Enamic; PEK: Pekkton; N: non-abraded surface; and A: abraded surface).

SEM images of material wear surface areas are shown in Figure 4. The occlusal surface image of VBM showed debris caused by wear and partial spallation, which produced a crater-like appearance. In the abraded area, large cracks and minor pitting were observed perpendicular to the sliding direction. YAP showed a large number of fragments, wear debris particles, partial spallation, and step-like fracturing of material along the sliding direction in the abraded area. MZD showed parallel grooves along the wear tracks, but generally smooth surfaces. ENA showed distinctive grooves, numerous microcracks, and pitted surfaces. PEK had the smoothest surface, with no cracks, spalling, pitting, or fractures.

## 4. Discussion

Tooth wear in the oral cavity is a complex process caused by many factors, such as the abrasive nature of food, parafunctional habits, antagonistic materials, enamel thickness and hardness, neuromuscular force, and chewing patterns [28,29,30]. Excessive wear can lead to the damage of occluding surfaces, loss of vertical dimensions of occlusions, functional path changes of masticatory movements, and fatigue of masticatory muscles [31]. It is desirable that the wear characteristics of restorative materials match those of natural teeth to protect opposing tooth surfaces and minimise occlusal disturbances [32,33]. In recent years, as aesthetic demands have increased, dental CAD/CAM systems in combination with machinable CAD/CAM materials have been used to produce restorations with excellent physical properties [14]. In the present study, the effects of cost-effective and time-saving CAD/CAM polymeric resins on primary tooth wear were investigated in order to suggest an innovative treatment method for restoring primary teeth.

The wear resistances of dental materials are important characteristics in clinical applications [8]. The physical properties of enamel, parafunctional habits, eating habits, and antagonist materials have all been reported to affect clinical wear [28,29,30,34]. Certainly, clinical studies provide the best means of establishing tooth wear, but they are costly and time consuming, and are disadvantaged by a lack of control over important variables such as chewing forces and environmental factors [35]. The results of in vitro studies are usually difficult to interpret directly into clinical practice, but these studies are cost effective and highly effective at achieving experimental goals [36]. The two-axes wear test device used in the present study reproduces the closure movement of the mandible during mastication by performing a sliding motion after occlusal contact [25]. The main types of wear occurring in the oral cavity are abrasive wear (two-body wear and three-body wear), fatigue wear, and corrosive wear [37]. In the present study, two-body wear tests were performed during which two surfaces were rubbed together without an intervening slurry of abrasive particles as a third medium [31]. In previous studies, when standardised antagonists of cusp shape and size (radius of 0.6 mm) were compared with natural and non-standardised antagonists, standardisation resulted in significantly different results for both antagonists and restorative materials, and did not reduce variations in resulting wear [38]. Thus, in the present study, tooth enamel cusp was not standardised. Several quantitative analysis methods of measuring the in vitro wear of dental materials have been described, which include measuring the surface roughness of worn specimens [39], specimen thickness differences before and after wear [40], and weight losses of worn specimens [41]. Heintze et al. [42] compared the abilities of 3D laser, mechanical, and optical methods to analyse the wear of dental materials, and concluded that all three methods were suitable, but that the laser-based method was faster and easier to use. In the present study, errors associated with the use of replicas were eliminated because volume losses were directly measured in antagonists and material specimens, rather than through indirect techniques using cast replicas [43], and 3D wear was measured using a non-contact scanner, which has been shown to be more effective and accurate [43]. Furthermore, the weight and volume losses of material specimens were measured to obtain objective data on material wear [25].

In the present study, CAD/CAM polymeric resins—that is, PMMA-based materials, hybrid ceramics, and high performance thermoplastic PEKK—were selected. Previous studies have shown that FDPs made from CAD/CAM polymeric resins have better fracture resistance than those produced from manually polymerised resins, and that they are less affected by ageing than polymerised resins and glass ceramics [3,44]. Also, CAD/CAM polymeric resins have colour stabilities and mechanical properties (e.g., flexural strengths) similar to those glass ceramics [8,45]. Therefore, CAD/CAM polymeric resins are regarded as being suitable for long-term restorations and as alternatives to glass ceramics in some patients [8]. 

In terms of antagonist wear, VBM and YAP (both PMMA-based materials) were found to cause more wear than MZD and PEK, which contrasts with the results of previous studies [2]. ENA, a hybrid ceramic, showed the second highest wear, and this high antagonist wear of ENA concurs with that found previously [46]. These results were explained by SEM images, which showed the presence of wear debris and rough surfaces on the worn surfaces of VBM, YAP, and ENA. Wear debris created during the wear process may be embedded between sliding surfaces, which not only increases the contact area, but also acts as a wear medium and generates a three-body wear mechanism and greater antagonist wear [47,48]. 

The high-performance polymer PEK showed the greatest material wear, while the hybrid ceramic ENA showed the least. The volume losses and weight losses of tested materials showed similar patterns. In general, material wear is closely related to mechanical properties, and increasing the flexural strength and hardness reduced the material abrasion [49,50]. Dupriez et al. [47] stated that material surface hardness and fracture resistance are essential for predicting material abrasion resistance, whereas Ferracane [50] reported that flexural strength and surface hardness are useful for predicting the abrasion degrees of composite resins. In the present study, material wear was smaller for MZD and ENA, which are hybrid composites with higher flexural strengths than VBM and YAP (both PMMA-based CAD/CAM materials with low flexural strengths). In addition, the wear resistance of composite resin teeth was found to be greater than that of acrylic resin teeth, which concurs with the findings of previous studies on various artificial teeth against enamel antagonists [31,51,52]. This finding can be explained by composite material composition and the presence of interpenetrating microstructures [51,52]. During wear testing, inorganic fillers distributed in MZD can increase the overall wear resistance by protecting the matrix, and nanosized fillers and reduced interparticle spacings also improve abrasion resistance [21]. Unlike traditional composites, which are continuous only in the matrix phase, ENAs with 3D interconnected dual network structures exhibited improved resistance to distinct modes of damage and have better physical properties than conventional composites [11,53]. These effects were supported by our SEM images, as MZD and ENA exhibited smoother wear surfaces than VBM or YAP [31]. On the other hand, although the flexural strength of PEK was higher than that of the other four materials examined, it also exhibited the greatest wear, which contradicts the manufacturer’s claim. However, VBM, YAP, and PEK have low elastic moduli, and thus can be easily deformed and irregularly displaced by stress.

Tooth morphological differences also affect wear rates [2], and dental material and lost dental tissue should possess similar mechanical properties to ensure restoration longevity and functional compatibility [54]. The limitation of this study is that primary tooth wear and severe, dynamic conditions in the oral cavity, such as temperature variations, pH fluctuations, and microbiota, were not investigated [54,55,56]. In addition, since the material specimens were not polished after CAM processing, it is unreasonable to directly compare the results with the clinical situation. Thus, clinical evaluation ought to be performed to confirm the results obtained in the present study.

## 5. Conclusions

Within the limits of this study, the volumetric and weight losses of materials exhibited similar patterns. PEK caused the least antagonist wear, but the greatest material wear. VBM and ENA caused significantly more antagonist wear than PEK, and ENA showed significantly less material weight loss than PEK. Since the wear patterns of antagonists and materials differed, it is evident that appropriate restorative materials selection should be based on considerations of specific clinical situations.

## Figures and Tables

**Table 1:** Characteristics of products used.

Type	Brand	Abbreviation	Composition	Flexural Strength (MPa)	Modulus of Elasticity (GPa)	Manufacturers
PMMA-based CAD/CAM materials	Vipi Block Monocolor	VBM	High cross-linked polymethylmethacrylate (PMMA)	100	2.2	Dental VIPI Ltda, Sao Paulo, Brazil
	Yamahachi PMMA	YAP	Polymethymetacrylate (PMMA)	N/A	N/A	Yamahachi Dental, Aichi, Japan
Resin nano ceramic	Mazic Duro	MZD	20 wt % of reinforced matrix and 80 wt % ceramic nanofillers	219.26	13.2	Vericom, Anyang, Korea
Hybrid ceramic	Vita Enamic	ENA	14 wt % acrylate–polymer and 86 wt % of fine structure feldspar ceramic	150–160	30	VITA Zahnfabrik, Bad Säckinge, Germany
High performance polymer	Pekkton	PEK	Polyeterketoneketone (PEKK)	200	5.1	Cendres + Métaux, SA, Switzerland

**Table 2:** Means and standard deviations (SDs) of volumetric losses of antagonists (V_a_), materials (V_m_), antagonists and materials (V_t_), and the weight losses of materials (V_w_) after wear.

Mean ± SD													
Group	n	V_a_ (mm^3^)			V_m_ (mm^3^)			V_t_ (mm^3^)			V_w_ (g)		
VBM	10	1.4261	±	1.6156 ^a^	1.0306	±	0.8135 ^a^	2.4567	±	1.9720 ^a^	0.0005	±	0.0002 ^ab^
YAP	10	1.2833	±	1.9111 ^ab^	0.8857	±	0.5807 ^a^	2.1690	±	1.7496 ^a^	0.0006	±	0.0005 ^ab^
MZD	10	0.7260	±	0.5786 ^ab^	0.7432	±	0.6296 ^a^	1.4692	±	0.6006 ^a^	0.0005	±	0.0002 ^ab^
ENA	10	1.3983	±	0.9264 ^a^	0.7197	±	0.4958 ^a^	2.1180	±	1.2189 ^a^	0.0003	±	0.0003 ^a^
PEK	10	0.2621	±	0.2707 ^b^	1.7617	±	1.4097 ^a^	2.0238	±	1.4974 ^a^	0.0008	±	0.0003 ^b^
*p*	-	0.021			0.682			0.900			0.024		

Note: Values followed by the same letter were not significantly different, as determined by the Mann–Whitney U-test with Bonferroni’s correction (*p* < 0.05/10 = 0.005). VBM: Vipi Block Monocolor; YAP: Yamahachi PMMA; MZD: Mazic Duro; ENA: Vita Enamic; and PEK: Pekkton.

## References

[1] Lauvahutanon S., Takahashi H., Shiozawa M., Iwasaki N., Asakawa Y., Oki M., Finger W.J., Arksornnukit M. (2014). Mechanical properties of composite resin blocks for CAD/CAM. Dent. Mater. J..

[2] Wimmer T., Huffmann A.M., Eichberger M., Schmidlin P.R., Stawarczyk B. (2016). Two-body wear rate of PEEK, CAD/CAM resin composite and PMMA: Effect of specimen geometries, antagonist materials and test set-up configuration. Dent. Mater..

[3] Alt V., Hannig M., Wostmann B., Balkenhol M. (2011). Fracture strength of temporary fixed partial dentures: CAD/CAM versus directly fabricated restorations. Dent. Mater..

[4] Barizon K.T., Bergeron C., Vargas M.A., Qian F., Cobb D.S., Gratton D.G., Geraldeli S. (2014). Ceramic materials for porcelain veneers: Part II. Effect of material, shade, and thickness on translucency. J. Prosthet. Dent..

[5] Zahran M., El-Mowafy O., Tam L., Watson P.A., Finer Y. (2008). Fracture strength and fatigue resistance of all-ceramic molar crowns manufactured with CAD/CAM technology. J. Prosthodont..

[6] Sripetchdanond J., Leevailoj C. (2014). Wear of human enamel opposing monolithic zirconia, glass ceramic, and composite resin: An in vitro study. J. Prosthet. Dent..

[7] Carvalho A.O., Bruzi G., Giannini M., Magne P. (2014). Fatigue resistance of CAD/CAM complete crowns with a simplified cementation process. J. Prosthet. Dent..

[8] Stawarczyk B., Özcan M., Trottmann A., Schmutz F., Roos M., Hämmerle C. (2013). Two-body wear rate of CAD/CAM resin blocks and their enamel antagonists. J. Prosthet. Dent..

[9] Nguyen J.F., Ruse D., Phan A.C., Sadoun M.J. (2014). High-temperature-pressure polymerized resin-infiltrated ceramic networks. J. Dent. Res..

[10] Awada A., Nathanson D. (2015). Mechanical properties of resin-ceramic CAD/CAM restorative materials. J. Prosthet. Dent..

[11] Della Bona A., Corazza P.H., Zhang Y. (2014). Characterization of a polymer-infiltrated ceramic-network material. Dent. Mater..

[12] Spitznagel F.A., Horvath S.D., Guess P.C., Blatz M.B. (2014). Resin bond to indirect composite and new ceramic/polymer materials: A review of the literature. J. Esthet. Restor. Dent..

[13] Zhi L., Bortolotto T., Krejci I. (2016). Comparative in vitro wear resistance of CAD/CAM composite resin and ceramic materials. J. Prosthet. Dent..

[14] Coldea A., Swain M.V., Thiel N. (2013). Mechanical properties of polymer-infiltrated-ceramic-network materials. Dent. Mater..

[15] Stawarczyk B., Jordan P., Schmidlin P.R., Roos M., Eichberger M., Gernet W., Keul C. (2014). PEEK surface treatment effects on tensile bond strength to veneering resins. J. Prosthet. Dent..

[16] Schwitalla A., Müller W.D. (2013). PEEK dental implants: A review of the literature. J. Oral Implantol..

[17] Tannous F., Steiner M., Shahin R., Kern M. (2012). Retentive forces and fatigue resistance of thermoplastic resin clasps. Dent. Mater..

[18] Fuhrmann G., Steiner M., Freitag-Wolf S., Kern M. (2014). Resin bonding to three types of polyaryletherketones (PAEKs)-durability and influence of surface conditioning. Dent. Mater..

[19] Seligman D.A., Pullinger A.G., Solberg W.K. (1988). The prevalence of dental attrition and its association with factors of age, gender, occlusion, and TMJ symptomatology. J. Dent. Res..

[20] Mahoney E., Holt A., Swain M., Kilpatrick N. (2000). The hardness and modulus of elasticity of primary molar teeth: An ultra-micro-indentation study. J. Dent..

[21] Mair L.H., Stolarski T.A., Vowles R.W., Lloyd C.H. (1996). Wear: Mechanisms, manifestations and measurement. Report of a workshop. J. Dent..

[22] Proffit W.R., Fields H.W. (1983). Occlusal forces in normal- and long-face children. J. Dent. Res..

[23] Sales-Peres S.H., Sales-Peres A.C., Marsicano J.A., Carvalho C.A., Carvalho F.S., Lauris J.R., Sales-Peres A. (2011). The relationship between tooth wear in the primary and permanent dentitions. Community Dent. Health.

[24] Shah P.V., Lee J.Y., Wright J.T. (2004). Clinical success and parental satisfaction with anterior preveneered primary stainless steel crowns. Pediatr. Dent..

[25] Choi J.W., Bae I.H., Noh T.H., Ju S.W., Lee T.K., Ahn J.S., Jeong T.S., Huh J.B. (2016). Wear of primary teeth caused by opposed all-ceramic or stainless steel crowns. J. Adv. Prosthodont..

[26] Lazaridou D., Belli R., Krämer N., Petschelt A., Lohbauer U. (2015). Dental materials for primary dentition: Are they suitable for occlusal restorations? A two-body wear study. Eur. Arch. Paediatr. Dent..

[27] D’Arcangelo C., Vanini L., Rondoni G.D., Pirani M., Vadini M., Gattone M., De Angelis F. (2014). Wear properties of a novel resin composite compared to human enamel and other restorative materials. Oper. Dent..

[28] Johansson A., Haraldson T., Omar R., Kiliaridis S., Carlsson G.E. (1993). An investigation of some factors associated with occlusal tooth wear in a selected high-wear sample. Eur. J. Oral Sci..

[29] Kim S.K., Kim K.N., Chang I.T., Heo S.J. (2001). A study of the effects of chewing patterns on occlusal wear. J. Oral Rehabil..

[30] Johansson A., Kiliaridis S., Haraldson T., Omar R., Carlsson G.E. (1993). Covariation of some factors associated with occlusal tooth wear in a selected high-wear sample. Eur. J. Oral Sci..

[31] Ghazal M., Hedderich J., Kern M. (2008). Wear of feldspathic ceramic, nano-filled composite resin and acrylic resin artificial teeth when opposed to different antagonists. Eur. J. Oral Sci..

[32] Willems G., Lambrechts P., Lesaffre E., Braem M., Vanherle G. (1993). Three-year follow-up of five posterior composites: SEM study of differential wear. J. Dent..

[33] Cha H.S., Lee Y.K., Lim B.S., Rhee S.H., Yang H.C. (2004). Evaluation of wear resistance of dental resin composites with a 3D profilometer. J. Biomed. Mater. Res. Part B Appl. Biomater..

[34] Rosentritt M., Siavikis G., Behr M., Kolbeck C., Handel G. (2008). Approach for evaluating the significance of laboratory simulation. J. Dent..

[35] Condon J.R., Ferracane J.L. (1997). In vitro wear of composite with varied cure, filler level, and filler treatment. J. Dent. Res..

[36] Lee A., He L.H., Lyons K., Swain M.V. (2012). Tooth wear and wear investigations in dentistry. J. Oral Rehabil..

[37] Mukatash-Nimri G.E. (2015). Wear mechanisms and wear investigations of dental materials; A review of the literature. Arch. Oral Dent. Res..

[38] Krejci I., Albert P., Lutz F. (1999). The influence of antagonist standardization on wear. J. Dent. Res..

[39] De Gee A.J., Pallav P., Davidson C.L. (1986). Effect of abrasion medium on wear of stress-bearing composites and amalgam in vitro. J. Dent. Res..

[40] Teixeira E.C., Thompson J.L., Piascik J.R., Thompson J.Y. (2005). In vitro toothbrush-dentifrice abrasion of two restorative composites. J. Esthet. Restor. Dent..

[41] Prakki A., Cilli R., Amarante de Araújo P., Navarro M.F., Mondelli J., Mondelli R.F. (2007). Effect of tooth-brushing abrasion on weight and surface roughness of pH-cycled resin cements and indirect restorative materials. Quintessence Int..

[42] Heintze S.D., Cavalleri A., Forjanic M., Zellweger G., Rousson V. (2006). A comparison of three different methods for the quantification of the in vitro wear of dental materials. Dent. Mater..

[43] Delong R. (2006). Intra-oral restorative materials wear: Rethinking the current approaches: How to measure wear. Dent. Mater..

[44] Stawarczyk B., Ender A., Trottmann A., Özcan M., Fischer J., Hämmerle C.H.F. (2012). Loadbearing capacity of CAD/CAM milled polymeric three-unit fixed dental prostheses: Effect of aging regimens. Clin. Oral Investig..

[45] Chaysuwan D., Sirinukunwattana K., Kanchanatawewat K., Heness G., Yamashita K. (2011). Machinable glass-ceramics forming as a restorative dental material. Dent. Mater. J..

[46] Stawarczyk B., Liebermann A., Eichberger M., Güth J.F. (2015). Evaluation of mechanical and optical behavior of current esthetic dental restorative CAD/CAM composites. J. Mech. Behav. Biomed. Mater..

[47] Dupriez N.D., von Koeckritz A.K., Kunzelmann K.H. (2015). A comparative study of sliding wear of nonmetallic dental restorative materials with emphasis on micromechanical wear mechanisms. J. Biomed. Mater. Res. Part B Appl. Biomater..

[48] Ratledge D.K., Smith B.G., Wilson R.F. (1994). The effect of restorative materials on the wear of human enamel. J. Prosthet. Dent..

[49] Peutzfeldt A., Asmussen E. (1992). Modulus of resilience as predictor for clinical wear of restorative resins. Dent. Mater..

[50] Ferracane J.L. (2013). Resin-based composite performance: Are there some things we can’t predict?. Dent. Mater..

[51] Ghazal M., Yang B., Ludwig K., Kern M. (2008). Two-body wear of resin and ceramic denture teeth in comparison to human enamel. Dent. Mater..

[52] Hirano S., May K.B., Wagner W.C., Hacker C.H. (1998). In vitro wear of resin denture teeth. J. Prosthet. Dent..

[53] El Zhawi H., Kaizer M.R., Chughtai A., Moraes R.R., Zhang Y. (2016). Polymer infiltrated ceramic network structures for resistance to fatigue fracture and wear. Dent. Mater..

[54] Ahmed N., Zafar M.S. (2014). Effects of wear on hardness and stiffness of restorative dental materials. Life Sci. J..

[55] Muhammad R., Ahmed N., Roy A., Silberschmidt V.V. (2012). Turning of advanced alloys with vibrating cutting tool. Solid State. Phenom..

[56] Rizvi A., Zafar M.S., Farid W.M., Gazal G. (2014). Assessment of Antimicrobial Efficacy of MTAD, Sodium Hypochlorite, EDTA and Chlorhexidine for Endodontic Applications: An In vitro Study. Middle East. J. Sci. Res..

